# Supplementary material for: Influence of Living Arrangements and Eating Behavior on the Risk of Metabolic Syndrome: A National Cross-Sectional Study in South Korea
Source: Int J Environ Res Public Health. 2019 Mar 14;16(6):919. doi: 10.3390/ijerph16060919 (PMC6466134; doi:10.3390/ijerph16060919)
Supplement: Supplementary file 1 [file ijerph-16-00919-s001.pdf]

Supplementary 1. Sociodemographic and health-related characteristics[1] of living arrangements and eating behavior for each age group.

	<65 years					≥65 years				
	LW×EW[2] (*n* = 10,765)	LW×EA (*n* = 363)	LA×EW (*n* = 467)	LA×EA (*n* = 165)	*p*	LW×EW (*n* = 3,099)	LW×EA (*n* = 283)	LA×EW (*n* = 307)	LA×EA (*n* = 566)	*p*
Sex										
Male	3906 (49.7)	112 (40.6)	234 (64.0)	63 (50.1)	<0.01	1447 (51.3)	97 (35.5)	75 (24.8)	106 (18.9)	<0.01
Female	5907 (50.3)	235 (59.4)	233 (36.0)	102 (49.9)		1422 (48.7)	176 (64.5)	232 (75.2)	460 (81.1)	
Income										
Q1	856 (8.1)	50(11.8)	96 (2.5)	81 (46.4)	<0.01	1182 (41.4)	99 (42.0)	226 (71.8)	449 (80.4)	<0.01
Q2	2394 (24.3)	91 (25.7)	136 (24.0)	47 (31.6)		846 (28.8)	86 (8.5)	56 (4.5)	83 (14.7)	
Q3	3124 (32.1)	90 (28.4)	119 (30.3)	23 (13.3)		481 (17.8)	58 (20.5)	16 (6.5)	19 (3.2)	
Q4	3394 (35.5)	114 (34.1)	113 (26.1)	14 (8.7)		340 (12.0)	23 (10.3)	9 (3.0)	10 (1.7)	
Education										
Elementary	892 (7.3)	59 (13.7)	81 (10.6)	64 (31.4)	<0.01	1470 (57.1)	169 (73.2)	207 (72.0)	395 (79.4)	<0.01
Middle	866 (8.2)	44 (13.0)	45 (7.1)	31 (20.0)		378 (14.5)	30 (11.1)	31 (11.6)	42 (8.9)	
High school	3607 (42.2)	123 (38.7)	144 (36.5)	32 (29.2)		464 (18.5)	26 (12.5)	35 (13.4)	40 (8.6)	
College or more	3642 (42.4)	98 (34.6)	167 (45.8)	27 (19.4)		261 (9.9)	9 (3.2)	9 (3.0)	15 (3.0)	
Employed, yes	5991 (64.4)	175 (52.3)	324 (70.7)	75 (48.9)	<0.01	873 (9.6)	65 (25.7)	83 (25.8)	128 (23.0)	<0.01
BMI, kg/m^2										
<18.0	258 (2.8)	6 (2.8)	11(2.6)	3 (2.0)	0.46	77 (2.8)	7 (3.5)	3 (1.0)	12 (2.0)	0.30
18.0–22.9	4064 (42.3)	148 (45.4)	175 (38.6)	58 (35.2)		1005 (35.1)	98 (37.5)	105 (33.4)	210 (38.3)	
23.0–24.9	2087 (21.4)	72 (22.7)	100 (22.5)	51 (30.0)		716 (26.7)	61 (20.5)	73 (25.2)	128 (23.9)	
≥25.0	3127 (33.5)	109 (29.1)	167 (36.2)	53 (32.8)		992 (35.4)	99 (38.5)	120 (40.4)	198 (35.6)	
Current smoking, yes	1798 (23.8)	48 (16.0)	152 (39.0)	46 (33.5)	<0.01	287 (11.5)	29 (11.4)	32 (2.2)	46 (9.3)	0.69
Lifelong experience of alcohol consumption, yes	8535 (93.0)	291 (91.2)	432 (96.3)	140 (90.2)	0.01	1994 (76.3)	180 (74.1)	193 (67.8)	302 (59.2)	<0.01
Walking hours/week	4.62 ± 0.10	5.26 ± 0.44	5.10 ± 0.41	5.09 ± 0.67	0.28	4.69 ± 0.16	5.14 ± 0.68	4.48 ± 0.43	4.12 ± 0.38	0.50
Eating out, n (%)										
Almost everyday	4537 (49.4)	52 (20.5)	272 (66.2)	19 (15.6)	<0.01	294 (11.0)	7 (2.9)	65 (21.1)	20 (3.8)	<0.01
Less than once a day	5455 (50.6)	293 (79.5)	193 (33.8)	146 (84.4)		2575 (89.0)	266 (97.1)	242 (78.9)	546 (96.2)	
Regularity of meals[3]										
Breakfast	6041 (57.6)	272 (74.9)	189 (33.3)	141 (78.8)	<0.01	2707 (93.5)	259 (95.5)	249 (11.9)	537 (46.0)	<0.01
Lunch	8810 (89.7)	301 (85.9)	413 (88.1)	155 (93.0)	0.17	2659 (92.4)	255 (8.6)	242 (5.4)	515 (91.5)	<0.01
Dinner	8714 (88.5)	313 (88.9)	387 (81.0)	154 (94.9)	<0.01	2769 (96.2)	261 (95.8)	281 (90.8)	538 (94.2)	0.01

≥3 meals[4]	5191 (49.3)	238 (65.4)	149 (25.4)	128 (71.8)	<0.01	2455 (84.5)	232 (86.0)	182 (56.6)	479 (84.7)	<0.01
<3 meals	4622(50.7)	109 (34.6)	318 (74.6)	37 (28.2)		414 (15.5)	41 (14.0)	125 (43.4)	87 (15.3)	
Eat alone										
Breakfast alone	2335 (34.4)	347 (100.0)	171 (72.4)	165 (100.0)		422 (17.4)	273 (100.0)	215 (81.7)	566 (100.0)	
Lunch alone	2577 (25.5)	347 (100.0)	119 (25.2)	165 (100.0)		718 (28.2)	273 (100.0)	66 (28.3)	566 (100.0)	
Dinner alone	1201 (13.3)	347 (100.0)	249 (53.0)	165 (100.0)		267 (11.0)	273 (100.0)	216 (73.0)	566 (100.0)	
Living alone	-	-	467 (100.0)	165 (100.0)				307 (100.0)	566 (100.0)	

[1] Continuous variables are presented as mean ± SE and discrete variables as n (%). [2] LW×EW: Living with others and eating with others; LW×EA: Living with others and eating alone; LA×EW: Living alone and eating with others; LA×EA: Living alone and eating alone. [3] Frequencies of those who have each meal 5–7 times a week. [4] Frequencies of those who have 3 meals 5–7 times a week.

Supplementary 2. Measures of risk factors for metabolic syndrome for each age group[1].

	<65 years					≥65 years				
	**LW×EW[2] (*n* = 10,765)**	**LW×EA (*n* = 363)**	**LA×EW (*n* = 467)**	**LA×EA (*n* = 165)**	***p***	**LW×EW (*n* = 3,099)**	**LW×EA (*n* = 283)**	**LA×EW (*n* = 307)**	**LA×EA (*n* = 566)**	***p***
Waist circumference (cm)	82.0 ± 0.7	82.1 ± 1.4	82.6 ± 1.1	83.8 ± 1.6	0.55	85.1 ± 1.0	84.8 ± 1.5	86.9 ± 1.3	85.4 ± 1.2	0.41
Triglycerides (mg/dl)	154.9 ± 12.3	143.3 ± 16.7	170.2 ± 16.0	169.7 ± 18.3	0.28	161.3 ± 9.9	156.9 ± 13.1	151.4 ± 13.5	153.9 ± 12.9	0.77
HDL cholesterol (mg/dl)	51.3 ± 0.7	51.5 ± 1.7	51.8 ± 1.1	48.7 ± 1.5	0.21	46.8 ± 1.3	46.7 ± 1.7	48.5 ± 2.1	46.6 ± 1.6	0.72
Systolic blood pressure (mmHg)	115.8 ± 1.0	119.2 ± 2.0	118.0 ± 1.5	118.5 ± 2.1	0.03	124.7 ± 2.0	129.8 ± 3.4	125.4 ± 2.9	128.6 ± 2.5	0.11
Diastolic blood pressure (mmHg)	76.2 ± 0.8	75.1 ± 1.5	78.2 ± 1.1	77.3 ± 1.4	0.10	71.1 ± 1.0	72.1 ± 1.6	73.7 ± 1.5	71.2 ± 1.4	0.32
Fasting blood glucose (mg/dl)	97.0 ± 1.6	97.4 ± 3.0	97.4 ± 2.2	102.5 ± 4.9	0.70	109.3 ± 2.9	108.8 ± 4.6	109.7 ± 3.9	107.7 ± 3.7	0.94
Number of risk factors for MetS	0.3 ± 0.1	1.6 ± 0.2	1.3 ± 0.2	1.7 ± 0.2	0.04	2.2 ± 1.2	2.2 ± 0.2	2.3 ± 0.3	2.3 ± 0.2	0.90

[1] Presented as mean ± SE. Adjusted for age, sex, income, education, smoking, alcohol consumption, physical activity, and total energy intake. [2] LW×EW: Living with others and eating with others; LW×EA: Living with others and eating alone; LA×EW: Living alone and eating with others; LA×EA: Living alone and eating alone; MetS: Metabolic syndrome.
